# Sensory defects in Necdin deficient mice result from a loss of sensory neurons correlated within an increase of developmental programmed cell death

**DOI:** 10.1186/1471-213X-6-56

**Published:** 2006-11-20

**Authors:** David Andrieu, Hamid Meziane, Fabienne Marly, Corinne Angelats, Pierre-Alain Fernandez, Françoise Muscatelli

**Affiliations:** 1 Institut de Biologie du Développement de Marseille Luminy, Campus de Luminy Case 907 13288 Marseille Cedex 09, France; 2 Institut Clinique de la Souris, 1 rue Laurent Fries, 67404 Illkirch Cedex, France

## Abstract

**Background:**

The human *NECDIN *gene is involved in a neurodevelopmental disorder, Prader-Willi syndrome (PWS). Previously we reported a mouse Necdin knock-out model with similar defects to PWS patients. Despite the putative roles attributed to Necdin, mainly from *in vitro *studies, its *in vivo *function remains unclear. In this study, we investigate sensory-motor behaviour in Necdin deficient mice. We reveal cellular defects and analyse their cause.

**Results:**

We report sensory differences in Necdin deficient mice compared to wild type animals. These differences led us to investigate sensory neuron development in Necdin deficient mouse embryos. First, we describe the expression pattern of Necdin in developing DRGs and report a reduction of one-third in specified sensory neurons in dorsal roots ganglia and show that this neuronal loss is achieved by E13.5, when DRGs sensory neurons are specified. In parallel, we observed an increase of 41% in neuronal apoptosis during the wave of naturally occurring cell death at E12.5. Since it is assumed that Necdin is a P75NTR interactor, we looked at the P75NTR-expressing cell population in Necdin knock-out embryos. Unexpectedly, Necdin loss of function has no effect on p75NTR expressing neurons suggesting no direct genetic interaction between Necdin and P75NTR in this context.

Although we exclude a role of Necdin in axonal outgrowth from spinal sensory neurons in early developmental stages; such a role could occur later in neuronal differentiation. Finally we also exclude an anti-proliferative role of Necdin in developing sensory neurons.

**Conclusion:**

Overall, our data show clearly that, in early development of the nervous system, Necdin is an anti-apoptotic or survival factor.

## Background

Molecules that orchestrate the cell cycle have to interact with extrinsic signals to trigger neural cell specification and differentiation [1,2]. Discovered 15 years ago, NECDIN (Neurally differentiated Embryonal Carcinoma-Derived proteIN, [3]), possesses all the characteristics of such a molecule as described in the literature [4,5]. Necdin belongs to the type II MAGE (Melanoma Antigen Gene Expression) gene family, all of whose members share a MAGE Homology Domain (MHD) of an as yet unknown function [5,6].

Different roles, mainly based on *in vitro *studies, have been assigned to Necdin. The mouse Necdin protein was first defined as a "growth-suppressor" that could facilitate the cell cycle exit and the maintenance of the neuronal postmitotic state [7-10]. A role in neuronal terminal differentiation, more specifically in neurite outgrowth and fasciculation was also supported by a number of studies [8,11-13]. This role is thought to implicate the Nerve Growth Factor (NGF) signalling pathway [8,11,12] and/or a signalling pathway involving centrosomal function and cytoskeletal rearrangement [13]. Supporting these findings, a large variety of Necdin partners have been revealed using yeast two-hybrids or Ras Rescue Systems. Necdin cytoplasmic interactors (FEZ1, BBS4, NEFA and Nuc which are both Ca2+ binding proteins), or nuclear interactors such as cell-cycle proteins (E2F1, E2F2, p53) and neurotrophic receptors (p75NTR, TrkA) have been characterized.

Interestingly, it is not only Necdin but also other members of the family such as NRAGE and MAGEH1 which interact with the death domain of the low affinity receptor to neurotrophin p75NTR [5,12,14,15].

As most of these data were obtained from *in vitro *experiments, their physiological relevance remained to be demonstrated. Thus, by focusing on the nervous system, we asked whether Necdin plays a unique role via a single signalling pathway or different roles, as suggested by the *in vitro *experiments, depending on the cell type and on the physiological context. Effectively, the Necdin expression profile in mouse is consistent with a role in neuronal differentiation including cell cycle arrest, neuronal maturation and later in maintaining a post-mitotic state [16].

The human *NECDIN *gene is deleted in the Prader-Willi syndrome (PWS) [17-19], a complex multigenic neurogenetic disease. PWS is mainly characterized by a transient infantile hypotonia, global development delay, hyperphagia leading to severe obesity and many other clinical features (hypogonadism, cognitive impairments, skin picking, daytime sleepiness, temperature instability, abnormal ventilatory responses...) [20].

Four different Necdin-null mouse models [18,19,21,22] have been generated in order to reveal the physiological and pathophysiological role of Necdin. At first glance, these mice show no obvious abnormal phenotype. Nevertheless, some phenotypic characteristics reveal striking parallels with the phenotypic manifestations in PWS patients: such as a high level of scraping, a particular cognitive profile [21] and, more importantly, depending on the genetic background, postnatal respiratory distress leading to lethality [18,21]. Up to now, the phenotype description of the Necdin knock-out (Ndn KO) model is still partial and further analysis could reveal other alterations similar to PW symptoms.

Finally, subtle morphological abnormalities were also reported suggesting that Necdin deficiency results in aberrant neuronal migration and affects axonal extension, arborization and fasciculation during development [13,23].

Here, in order to clarify the functional roles of Necdin in the cellular physiology of the nervous system, we investigate the sensory system. First, we explore the sensory-motor behaviour and reveal sensory deficits that might result from Necdin deficiency. Then, to address the function of Necdin at the cellular level we examine the integrity of Dorsal Root Ganglias (DRGs) in Necdin mutant embryos (Ndn KO) we have previously generated [21]. We analyse carefully the spatio-temporal expression pattern of Necdin in the developing sensory neurons. We observe a significative loss of TrkA and TrkC sensory neurons in Ndn KO compared to wild type; this loss is achieved at E13.5 dpc and remains at P0. We reveal an increase of caspase3-dependant apoptosis, before the peak of naturally occurring cell death. This cell death does not involve the P75NTR receptor.

We also investigate the axonal outgrowth in several structures innervated by the spinal sensory neurons in Necdin mutants.

## Results

### Phenotypic features suggest defects of sensory pathways

Phenotypical analysis is performed on 7 wild type and 9 mutant adult mice (3–4 months) (Table 1). Ndn KO mice had normal body weight and body temperature (Table 1), well-groomed coats, and normal body posture. Gross neurological examination of animals reveals no distinct signs of modified sensory or vestibular reflexes in mutants as assessed in simple tests of vision, audition, olfaction, touch sensitivity or righting reflex. Necdin mutants also show a normal reactivity to handling and a normal exploratory activity when exposed to a novel cage immediately following the transfer arousal test.

**Table 1:** Neurological evaluation

		***Wild type***	***Ndn-KO***
***Body weight (g)***		25.20 (0.55)	25.80 (1.75)
***Body temperature (°C)***		36.40 (1.03)	36.60 (0.60)
***String test (s)***		8.98 (20.62)	7.37 (2.66)
***Grip strength (s)***		7.90 (0.98)	7.67 (1.85)
***Rotarod-4 to 40 rpm in 5 min (s)***		117.50 (76.38)	90.50 (69.88)
***Beam walking (s)***	*Number of slips*	1.0 (0.25)	2 (2.40)
	*Latency (s)*	11.59 (4.10)	14.66 (2.39) *

Values indicated represent the median and, between brackets, the interquartile range. * p < 0.05, Mann-Whitney

We explored further the sensory-motor abilities and general behaviour of Necdin adult deficient mice using specific behavioural tests. Mutant mice had comparable level of spontaneous activity to wild types, as revealed by the distance travelled and number of rears in the open field test (Table 2). The number of entries and percentage of time spent in the center of the open field arena was also comparable between genotypes, suggesting that Necdin deletion has no effect on anxiety at least in this test. When analyzed for specific motor abilities, mutant mice performed as well as the wild type mice in the grip and string tests, showing that muscle strength and traction force are not affected in mutants (Table 1). On the other hand, the time spent in an accelerated rotarod was reduced and the latency to reach the goal platform in the beam walking test was significantly increased (p < 0.05; Table 1) in Necdin deficient mice as compared to wild type, suggesting motor coordination or balance deficits in mutants. Mutant mice also showed a higher tendency to slip off with the hind limbs that might be interpreted as altered proprioceptive sensitivity.

**Table 2:** Open Field

	***Wild type***	**Ndn-KO**
***Distance traveled (cm)***	12965(11228,13406)	13186((11704, 14717)
***Time spent in center (%)***	5.4 (4.9, 7.3)	6.3 (5.3,7.3)
***Entries in center (nb)***	78 (70, 94)	88 (85, 106)
***Rearings (nb)***	265 (219,275)	249 (188, 295)

Values indicated represent the median and, between brackets, the interquartile range.

Electrophysiological tests to evaluate innervation of peripheral targets and spinal cord reflexes, were performed on hindlimbs (Table 3). The sensory nerve conduction velocity (SNCV) is comparable between wild type and Necdin deficient mice. The compound muscle action potentials including the direct muscle response (M-wave) at the level of the gastrocnemius or plantar muscles, and the monosynaptic reflex response (H-wave) on the plantar muscle were also recorded. Amplitude and latency of the M-wave were not significantly different between mutant and wild type mice. On the other hand, the H-wave amplitude and the H/M amplitude ratio, recorded on the plantar muscle, were significantly higher in the mutant group (p < 0.05). The H-reflex amplitude and the H/M amplitude ratio have been used traditionally to evaluate spinal excitability. These data suggest an overall facilitation of the stretch reflex circuit (H-reflex) that could result from hyperexcitability of motoneurons and/or sensory neurons.

**Table 3:** EMG measurements

			***Wild type***	***Ndn KO***
***Sensory Nerve Conduction Velocity (m/s)***			38.5 (8.48)	40.00 (11.43)
***Plantar muscle CMAP***	M-wave	Latency (s)	1.65 (0.13)	1.58 (0.19)
		Amplitude (mV)	4.60 (4.98)	4.50 (1.90)
	H-wave	Latency (s)	4.60 (0.68)	4.30 (0.20)
		Amplitude (mV)	0.40 (0.18)	0.70 (0.20) *

Values indicated represent the median and, between brackets, the interquartile range. * p < 0.05, Mann-Whitney

Finally, the hot plate test was used to evaluate pain sensitivity and revealed significantly reduced withdrawal latency in mutant mice compared to wild type animals (p < 0.05; Table 4), thus suggesting reduced pain threshold in Necdin deficient mice.

**Table 4:** Pain sensitivity

	***Wild type***	***Ndn-KO***
***Hot plate latency (s)***	9.13 (2.08)	7.05 (1.23) *
***Tail flick latency (s)***	3.77 (0.98)	3.03 (0.28)

Values indicated represent the median and, between brackets, the interquartile range. * p < 0.05, Mann-Whitney

Taken together these data reveal significant defects in the sensory system.

### Expression of Necdin in developing DRGs

**Figure 1:**
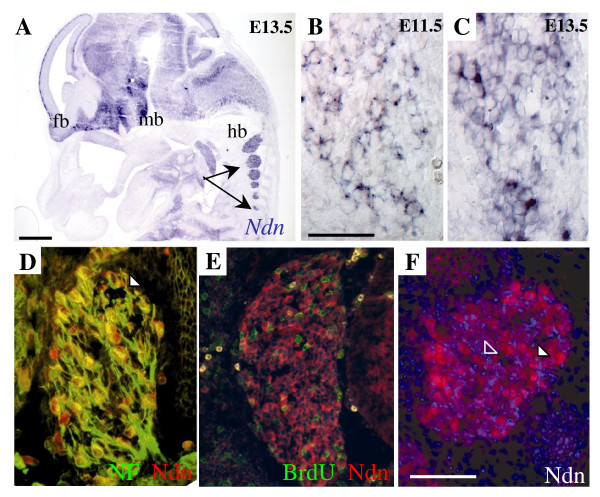
**Expression of Necdin in DRGs through development**. (A) *Ndn in situ *hybridization (ISH) on sagittal section at E13.5 displays expression in the central and peripheral nervous system, in particular in the forebrain (fb), the midbrain (mb), the hindbrain (hb) and in the DRGs (arrows). (B, C) *Ndn *mRNA is detected in developing sensory neurons at E11.5 (B) and E13.5 (C). At E12.5, Necdin protein is not expressed in progenitor cells but in post-mitotic neurons. (D) Sections through lumbar DRGs of E12.5 embryos co-labelled with anti-Necdin antibody (in red) and with anti-Neurofilament antibody (in green) reveal a co-localisation between both staining (in yellow). (E) Consecutive sections double-labelled with anti-Necdin antibody (in red) and anti-BrdU (in green) show no co-localisation of both markers. (F) Magnification of a lumbar DRG at E13.5 labelled by immunohistochemistry using anti-Necdin antibody in combination with Hæchst, to look at the subcellular localisation of Necdin. Necdin is detected in the cytoplasm and nucleus of cells. Arrowheads indicate examples of both weak and strong expression level of Necdin in sensory neurons that are readily observable at E13.5. Scale bar: 200 μm (A); 50 μm (B and C); 100 μm (D, E and F).

In order to detect histological abnormalities in lumbar DRGs that could be correlated to the sensory dysfunction in Ndn KO mice, we first analysed the morphology and the size of lumbar DRGs. At P0, comparing the volume of the first lumbar (L1) DRG between Ndn KO and wild type mice (WT), we observed a 37% reduction of the mutant L1 DRG volume (WT: 32 10^5 ^μm^3 ^(30 10^5^, 32.7 10^5^), n = 3; Ndn KO: 19.7 10^5 ^μm^3 ^(17.2 10^5^, 21.4 10^5^), n = 3). Since previously, we have shown that Necdin is expressed through embryogenesis in postmitotic neurons [16], we hypothesized that a defect during the ontogeny of sensory neurons in Necdin deficient mice might result in a loss or pronounced atrophy of sensory neurons resulting in a reduction of L1 DRGs volume in Necdin deficient mice. In developing DRGs, expression of *Necdin *transcript is first detected at E11.5 and progressively increases to E13.5, in accordance with an increasing number of neurons present in ganglia (Fig. 1A–C). At E13.5, using the thymidine analogue bromodeoxyuridine (BrdU), to label proliferative cells, we revealed the absence of Necdin expression in BrdU-positive cells (Fig. 1E); however we showed a colabelling of Necdin and Neurofilament (NF) by immunohistochemistry (IH) (Fig. 1D). Therefore, in developing DRGs, Necdin is clearly expressed in post-mitotic neurons and not in the progenitor cells as it has been shown in other brain structures [16]. Necdin protein is detected in the nucleus and the cytoplasm (Fig. 1F), as confirmed by confocal analysis (not shown).

**Figure 2:**
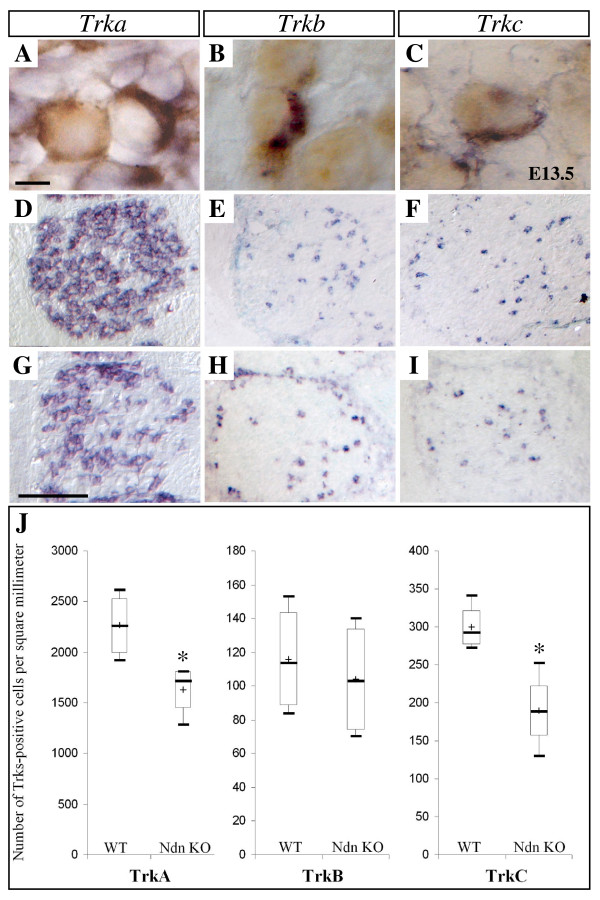
**Reduction of the number of TrkA- and TrkC-expressing cell in Necdin mutant DRGs**. (A-C) Transverse section of E13.5 embryos double-labelled with an anti-Necdin antibody and with the riboprobes of *Trka *(A) or *Trkb *(B) or *Trkc *(C) respectively. *In situ *hybridisation on sections of lumbar DRGs from E13.5 wild type (D-F) and Necdin mutant (G-I) embryos with the riboprobes *Trka *(D, G), *Trkb *(E, H) and *Trkc *(F, I). (J) Number of *Trk*-expressing neurons in lumbar DRGs at E13.5. Shown are the box-plots, describing the number of neurons per square millimetre for five ganglia from five embryos of each genotype (mutant or wild type). Significant loss of TrkA- and TrkC-subpopulations is observed. Statistical comparisons are made using the Mann-Whitney test; asterisks show differences that are statistically significant (*, p < 0.05). Scale bar: 100 μm (A-I).

In an attempt to determine which class of specified DRG neurons express Necdin, we examined the expression of the three tyrosine kinase receptors (Trk), TrkA, TrkB and TrkC; characterizing respectively NGF responding nociceptive neurons, BDNF responsive neurons and NT3 responsive proprioceptive neurons [24]. At E13.5, *In Situ *Hybridization using the riboprobes *Trka *(Fig. 2A), *Trkb *(Fig. 2B) and *Trkc *(Fig. 2C) combined with immunostaining using Necdin antibodies showed that all *Trkc*- and *Trkb*-expressing neurons, as well as a significantly high number (80%) of *Trka*-expressing neurons, co-expressed Necdin.

### Lack of Necdin causes embryonic loss of sensory neurons

With regard to Necdin expression, we examined more closely the TrkA, TrkB and TrkC sensory neuron subpopulations. As these populations are highly dynamic through gangliogenesis [25], we studied different embryonic stages from E11.5, when discernible DRGs are formed, to E13.5, when DRG neurons are specified. At all stages, *Trka*, *Trkb *and *Trkc *expressing cells were detected in mutant mice as shown at E13.5 (Fig. 2G–I). At E11.5 a quantitative analysis of each *Trk*-expressing cell subpopulation in lumbar DRGs (L1 to L5), revealed no difference between wild type and mutant embryos (data not shown). At E13.5, the morphological appearance and the volume of lumbar DRGs are similar in mutant and wild type embryos. However,, the pool of lumbar DRGs revealed a significant reduction of 26.2% in the density of *Trka*-expressing cells (WT: 2258 (1999, 2530), n = 5; Ndn KO: 1714 (1450, 1811), n = 5; *p < 0.05) (Fig. 2D,G,J) and a reduction of 37.8% in *Trkc*-positive neurons (WT: 292 (277, 321), n = 5; Ndn KO: 188 (157, 223), n = 5; *p < 0.05) (Fig. 2F,I,J)) in the mutant embryos. The density of *Trkb*-expressing neurons was not affected (WT: 113 (89, 143), n = 5; Ndn KO: 102 (74, 133), n = 5) (Fig. 2E,H,J).

At P0, in order to confirm the loss of sensory neurons in Ndn-KO DRGs, we compared the expression of TrkA in L1 DRGs specifically, between mutant and wild type neonates (WT: 2050 (1700, 2310), n = 3; Ndn KO: 1243 (1065, 1680), n = 3).

These data indicate that the abrogation of Necdin results in a partial loss of sensory neurons expressing TrkA and TrkC receptors. This loss occurs in early embryonic development and is maintained at birth. The number of TrkB-expressing neurons appears similar in mutant and wild type animals.

### Apoptotic cell death is increased in developing DRG neurons in Necdin mutant

**Figure 3:**
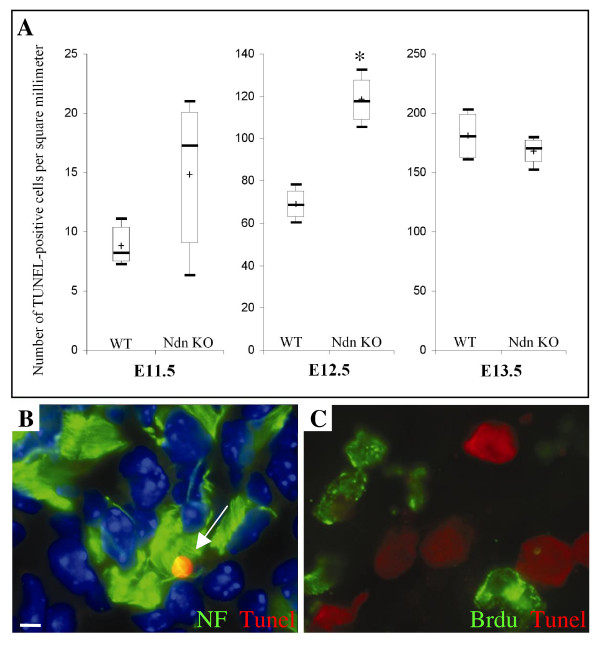
**Increase of apoptosis in the Necdin mutant DRGs**. (A) Time course of TUNEL staining in lumbar DRGs. Shown are the box-plots describing the number of TUNEL positive cells for lumbar DRGs; statistical comparisons were made using the Mann-Whitney test; asterisks show differences that are statistically significant (*, p < 0.05). (B) Double immunofluorescence labelling for TUNEL (in red) and Neurofilament (in green) in a lumbar ganglion of an E12.5 mutant embryo. In this figure, one immunopositive-cell for Neurofilament in the field whose nuclei are labelled by the apoptosis detection method (arrow). (C) Double-labelling for TUNEL (in red) and BrdU (in green) after 2 hr injection; co-labelling was never observed, indicating that precursors are not dying in the Necdin mutant animals. Scale bar: 10 μm (B and C).

As the physiological wave of cell death among DRGs neurons occurs mainly from E12.5 to E14.5 [26], we hypothesized that an increase of cell death could be responsible for the loss of sensory neurons in Necdin mutants. We quantified cells undergoing DNA fragmentation, using a TUNEL assay (terminal deoxynucleotide transferase (tdt)-mediated dUTP nick end labelling), in the lumbar region and at different developmental stages (E11.5, E12.5 and E13.5) (Fig. 3A). In wild type mice, we observed an increase of apoptosis in the lumbar region between E11.5 and E13.5, as previously reported [26]. In Necdin mutants, at E11.5 (Fig. 3A), we observed no significant difference with the wild type control littermate (WT: 8 (7, 10), n = 5; Ndn KO: 17 (9, 20), n = 3). However, at E12.5, we observed a significant 41% increase in the density of TUNEL positive cells in mutants compared to control DRGs (WT: 68 (62, 75), n = 4; Ndn KO: 117 (108, 127), n = 3; * p < 0.05) (Fig. 3A). Finally, at E13.5, the density of TUNEL positive cells was equivalent in both mutants and wild type (WT: 180 (163, 199), n = 3; Ndn KO: 170 (159, 177), n = 3). Double labelling using the TUNEL assay and immunostaining with anti-NF (Fig. 3B) revealed a colocalisation of both markers, indicating that dying cells are mainly post-mitotic. In accordance, TUNEL staining combined with BrdU labelling (Fig. 3C) indicated that progenitors are not affected.

Finally, in mutant mice, we compared serial sections marked either with FluoroJade, a marker of non specific neuronal degeneration [27], or with TUNEL staining. These two labellings, gave a similar, scattered pattern of stained neurons suggesting no increase of a necrotic non-apoptotic process in mutant DRGs (data not shown).

In conclusion, we observed an increase of apoptosis in Necdin mutant lumbar DRG mice at E12.5 precisely, just before the normal peak of cell death occurring at E13.5. This cell death is restricted to neurons and does not affect progenitors.

### The increase of apoptosis observed in early sensory neuron development of Necdin deficient mice is not dependent on p75NTR

Different studies suggest that Necdin could be involved in the signalling pathway mediated by p75NTR [12,15,16,22]. Interestingly, mice with a targeted deletion in p75NTR show a partial loss of sensory neurons in DRGs [28,29]. We propose that if the sensory neurons lost in Necdin mutant embryos are those expressing p75NTR, then we would expect, at E13.5, a reduction of the number of neurons expressing p75NTR in Necdin mutant mice.

**Figure 4:**
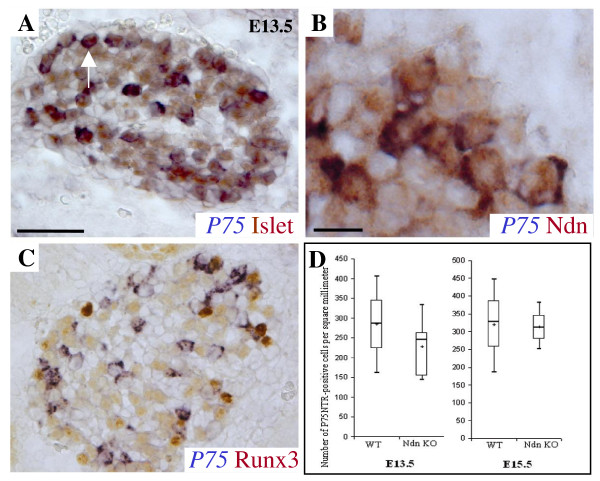
***In vivo*, cell death in Necdin mutant developing DRGs is not dependent on P75NTR**. (A-C) DRG sensory neurons at E13.5 on a transverse section are double-labelled with *p75NTR *mRNA and Islet1/2 (A), or with Necdin (B) or with Runx3 antibodies, a marker of proprioceptive sensory neurons (C). In each case, colabelling reveal a partial co-expression. (D) Quantification of the number of *p75NTR*-positive cells in mutant compared to the wild type at distinct developmental stage. Shown are the box-plots describing the numbers of *p75*-expressing cells per square millimetre at E13.5 and E15.5, and statistical comparisons were made using the Mann-Whitney test. Scale bar : 100 μm (A and C); 50 μm (B).

Initially, in wild type embryos, we clarified the expression pattern of P75NTR in sensory neurons. We performed three separate double labelling experiments with a *p75NTR *riboprobe and either an anti-Islet1/2 antibody (Fig. 4A), an anti-Necdin antibody (Fig 4B), or an anti-Runx3 antibody, labelling the TrkC expressing neurons (Fig. 4C). Colabelling results showed that *p75NTR *is expressed only in a subset of sensory neurons (Islet1/2-positive), in a subpopulation of Necdin expressing neurons and more particularly in 40% of proprioceptive (TrkC) neurons (Runx3-positive). Noticeably, an equivalent amount (37.8%) of *Trkc*-positive cells was lost in Necdin deficient mice.

We then compared the number of cells expressing *p75NTR *in the lumbar DRGs of wild type and Necdin mutant. At E13.5 (Fig. 4D), the quantification of the p75NTR-expressing cells did not reveal any difference between wild type and mutant lumbar DRGs (WT: 286 (223, 346), n = 3; Ndn KO: 246 (154, 262), n = 5). At E15.5, this quantification is similar (WT: 327 (257, 386), n = 3; Ndn KO: 312 (280, 346), n = 4) as at P0 (WT: 1816 (1730, 1880), n = 4; Ndn KO: 1833 (1813, 1949), n = 3).

Indeed, these results show that cell death observed in Necdin mutant DRGs does not affect specifically the p75NTR-expressing neurons and consequently does not directly involve the p75NTR-signalling pathway.

### Neurite outgrowth in developing sensory neurons of Necdin mutants

**Figure 5:**
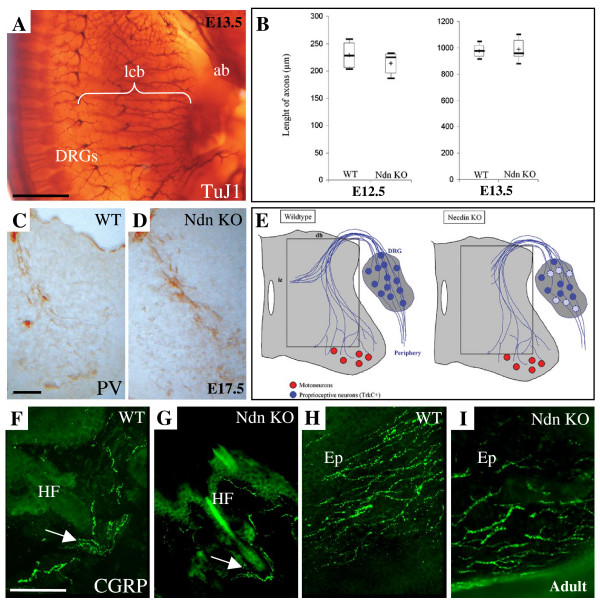
***In vivo *analysis of axonal growth in Necdin mutant embryos**. (A) Whole-mount immunostaining of E13.5 wild type and mutant embryos with TuJ1, a neuronal specific marker illustrating the parameter used for quantification (lcb). (B) Quantitative analysis of the length of the lateral cutaneous branches of the nerves innervating the trunk at E12.5 and E13.5. No significant differences in the length of spinal nerves were observed in mutant embryos compared to wild type. Six peripheral axon bundles, from five mice, for each genotype, were analyzed. Statistical analysis was carried out using the Mann-Whitney test. (C, D) Parvalbumin immunostaining on transverse section at E17.5 showing a decrease of Necdin mutant proprioceptive afferences (D) in spinal cord compared to wild type (C). (E) Representative scheme of defect observed in (C) and (D). (F-I) CGRP immunostaining on coronal section, at adulthood, showing a similar innervation pattern of TrkA fibers in the hindpaw pad (F-G) around an hair follicle or (H-I) in the epidermis under nail of the second toe in NdnKO (G, I) or wild type (F-H) mice. We notice a decrease in the number of fibers in Ndn KO. ab, anterior bud; Ep, epidermis; HF, hair follicle; lcb, lateral cutaneous branch. Scale bar: 500 μm (A); 100 μm (C, D and F-I).

To investigate whether Necdin is required for axonal elongation in sensory neurons as has been previously suggested, in the superior cervical ganglia at E18 [23], we examined the axonal outgrowth in wild type and mutant embryos in several structures innervated by the DRGs sensory neurons. First, we performed a whole-mount immunostaining using anti-β tubulin III (Tuj1) antibody, an axonal marker, to visualize the morphology and to measure the length of axons in mutants compared to wild type (Fig. 5A). We analyzed different nerves along the rostro-caudal axis. At E11.5, in Necdin mutant embryos, the brachial plexus, formed by the last three cervical and first two thoracic nerves, in the forelimb was appropriately patterned and all nerves emanating from it appear normal compared to wild type (data not shown). At E12.5 and E13.5, peripheral spinal nerves were analysed quantitatively by measuring the length of nerves (given in μm) innervating the trunk region. Statistical analysis did not reveal any difference between mutant and wild type neither at E12.5 (WT: 227 (206, 252), n = 4; Ndn KO: 224 (195, 230), n = 5) nor at E13.5 (WT: 979 (935, 1025), n = 7; Ndn KO: 959 (930, 1066), n = 9) (Fig. 5B). Thus, at early stages of development (E11.5 to E13.5) our data suggest that Necdin does not play a major role in axonal growth in those structures.

At E17.5, when sensory axons from DRG reach their targets, we examined whether axonal projections were correctly patterned. We performed immunohistochemistry with anti-Parvalbumin antibody (PV), which detects specifically proprioceptive (TrkC) afferent fibers entering the spinal cord [30] (Fig. 5C and 5D). Comparison of PV-immunoreactive fibers in control (Fig. 5C) and Necdin mutant mice (Fig. 5D) showed that axons extend correctly ventro-laterally through the gray matter from the dorsal column to the ventral horn. However, in mutant mice, PV-immunostaining showed a difference in the region of the intermediate spinal cord, close to a set of Islet-positive neurons located in the deep dorsal horn [30]. We clearly observed that afferent projections from the proprioceptive neurons are reduced in the intermediate spinal cord (Fig. 5E).

Finally at P0 and at adulthood, we examined the cutaneous innervation in the hindpads of mice, revealed by using immunofluorescence with an antibody for calcitonin gene-related product (CGRP), a marker of nociceptive (TrkA) afferent fibers. At P0 and adulthood, CGRP-immunoreactive fibers are found in the epidermis, around the hair follicles, in the upper dermis and around blood vessels as in wild type animals (Fig. 5F and 5H) and as previously described [31]. However, we noticed a reduction in the number of CGRP-immunoreactive fibers in mutant mice.

In conclusion, we did not observe any gross impairment of axonal outgrowth in early developmental stages (E11.5–E13.5) in our Ndn KO embryos. Later in development and in adult, we showed that PV-fibers and CGRP-fibers that express TrkC and TrkA respectively are correctly patterned; however we detected a reduction in the number of fibers.

**Figure 6:**
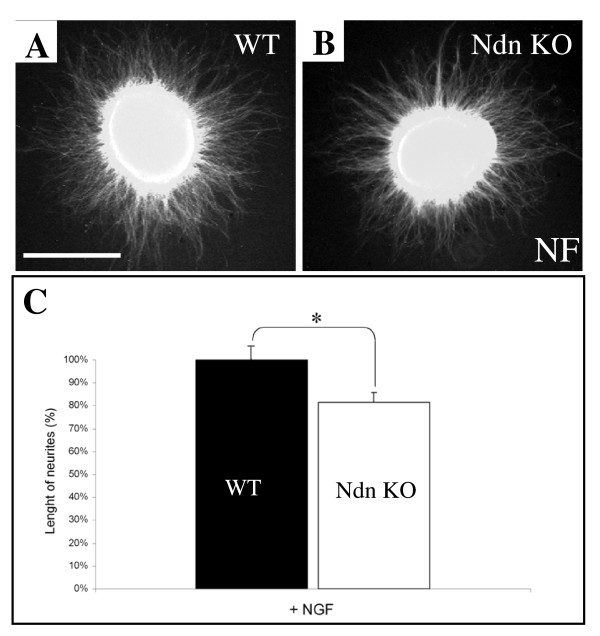
**Abrogation of Necdin imparts neuritic extension in explants DRGs**. (A, B) Photomicrographs of DRGs from E13.5 wild type (A), and Necdin mutant (B) embryos, cultured for 20 hr in the presence of NGF only (100 ng/ml). In three separate experiments, performed in four mutant and wild type lumbar DRGs, significant NGF effect was observed in explants derived from Necdin mutants (C). Scale bar: 100 μm (A and B).

Since we did not observe any gross impairment of axonal outgrowth in our Ndn KO mice although others have reported such defects, specifically *in vitro *[11], we then assessed *in vitro *the role of Necdin on neurite outgrowth as it has been shown in a different Ndn-KO mutant [22]. DRG explants isolated from E13.5 were cultured for 48 hours in the presence of either the neurotrophic factor NT3 or NGF. In the presence of NT3, DRG explants from wild type and KO extended neurites with similar length (data not shown). However, in the presence of NGF, a significant reduction of 18,3% (Fig. 6H) was observed in the length of neurites in Necdin mutant explants cultured, compared to the control (Fig. 6), suggesting that Necdin deficiency impairs the neurites extension induced by NGF signalling in DRGs explants.

## Discussion

Until now, Necdin function was investigated mainly by *in vitro *experiments, which suggested a role of Necdin during proliferation, in neuronal differentiation and/or apoptosis. Here, using a Necdin knock-out mouse model, we have first analysed the sensory-motor function in adult mutants and second investigated the *in vivo *cellular function of Necdin during the development of the mouse nervous system and in particular in sensory neurons of DRGs.

In this instance, DRGs are a system well adapted to study the cellular function of Necdin in the nervous system. In DRGs, neural progenitors coexist with differentiated sensory neurons throughout all of neurogenesis and the ontogeny of sensory neurons is well characterized in terms of origins, proliferation, determination, cell death and neuronal survival [26].

### Necdin prevents apoptosis in early developing sensory neurons

Thus Necdin deficiency impairs lumbar sensory neuron development between E11.5 and E13.5, resulting in a loss of sensory neurons persisting at P0 and at adulthood (FM personal data). During this time frame, we observed in our control mice a wave of naturally occurring cell death as previously described [26]. We revealed a significant 41% increase of cell death in lumbar Necdin mutant DRGs compared to wild type. This increase concerns post-mitotic neurons only and occurs at E12.5, just before the peak of naturally cell death, which is observed in normal mice at E13.5. This increase of cell death was confirmed by Caspase-3 staining and Hoechst incorporation (data not shown). Thus, abrogation of Necdin triggers an increase of early apoptosis via the Caspase-3 pathway.

Notably, this increase of apoptosis and subsequently the loss of sensory neurons observed in Necdin mutant are specific to the lumbar region. No defect is detected at the thoraco-brachial level (Additional File 1). However, at E13.5, a similar loss of sensory neurons was obtained in the primary sensory neurons of the trigeminal ganglia (Additional File 2).

**Figure 7:**
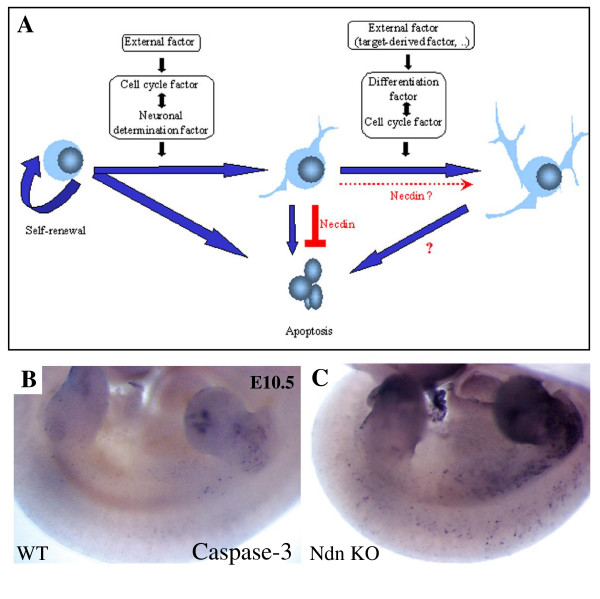
**Putative role of Necdin during development**. (A) Schematic representation of Necdin functions in nervous system *in vivo*. (B and C) Cell death in early embryos at E10.5 assessed by Caspase3-immunostaining in wild type (B) and mutant (C) embryos.

Clearly, at the cellular level, Necdin prevents apoptosis but the mechanism is unclear. Necdin could be an anti-apoptotic factor or equally it could be having a positive survival effect on the sensory neurons (Fig. 7A).

### Are specific neurotrophic factors and/or neurotrophic receptors involved in Necdin signalling pathway(s)?

The wave of cell death in normal developing DRGs, at E13.5, occurs before sensory axons have reached their targets and before neurons are actually exposed to target derived neurotrophic factors [32,33]. Thus, at E12.5, the role of Necdin is probably independent of neurotrophic factors provided by the target. However Necdin signaling pathway might depend on local action of neurotrophins or other growth factors.

The timing of neurotrophin/Trk signalling requirements and the cell types affected by the neurotrophin deficiencies have been well studied [26]. It is assumed that a single neurotrophic factor might promote the survival of a specific population of sensory neurons, although phenotypic comparisons among mice with different mutations have revealed a considerable overlap in terms of neurotrophin requirements [34]. The abrogation of Necdin results in a relatively selective loss of neurons in DRGs: TrkA and TrkC cell populations are reduced but not TrkB population. A simple interpretation of that might be that Necdin transduces a survival signal of NGF via TrkA (as previously proposed [22]) or/and of NT3 via TrkC. Considering that neurogenesis in developing DRGs is a dynamic process and the expression patterns of the different Trk proteins are also highly dynamic between E10.5 and E13.5, another interpretation is that the TrkA and TrkC populations present at E13.5 expressed a common receptor at an earlier stage of their development and required a common neurotrophic factor that could trigger Necdin function as a survival factor.

Another way to explain the mechanism, by which Necdin could act as an anti-apoptotic/survival factor, is to consider the p75NTR pathway. Previously, it has been shown that p75NTR interacts with Necdin [12] and a common signalling pathway involving Necdin and p75NTR has been proposed [15,22]. Furthermore, throughout development, Necdin and p75NTR expression partially overlaps in the post-mitotic territories [16]. However, we did not observe any reduction in the number of neurons expressing p75NTR in Necdin embryonic DRGs compared to wild type. These data suggest that there is no direct genetic interaction between p75NTR and Necdin to prevent the regulated cell death in post-mitotic developing sensory neurons.

### Cell proliferation is not increased in DRGs of Necdin deficient mice

Necdin has been demonstrated to function as a "growth suppressor" *in vitro*, since its ectopic expression induces an arrest of cell growth [7,8]. Considering such a role, we could hypothesize that the increase of cell death observed in DRGs from mutant mice might be subsequent to a previous increase of proliferation. Indeed these events are tightly linked and a compensation mechanism cannot be excluded [35]. An "anti-proliferative" role of Necdin should induce, in Necdin deficient DRGs, a reduction in the pool of post-mitotic generated neurons and an increase of the number of precursors and/or an increase in cell death. Three types of data do not support such a role in developing DRGs: 1) at E11.5, we did not observe any reduction of the number of Trks expressing cells, including the entire pool of post-mitotic neurons at this stage; 2) we performed BrdU labelling experiments at E13.5 to assess the amount of proliferation in DRGs and no difference was observed as checked by analysis on one representative lumbar DRG; 3) the increase of apoptosis observed at E12.5 is restricted to post-mitotic neurons and neither apoptosis nor necrosis of precursors were observed.

### The abrogation of Necdin does not disturb the axonal growth in early stages of neuronal differentiation

Altogether, the analysis of axonal growth made in Necdin mutant compared to wild type mice, suggests that, *in vivo*, Necdin is not involved in early axonal outgrowth. Later in development at E17.5, we observed a reduced innervation from the TrkC afferent fibers in the intermediate spinal cord, visualised by a Parvalbumin labelling. Thus this specific defect of the TrkC neurons might suggest an involvement of Necdin in axonal growth. However, we have shown that the lack of Necdin leads to a loss of TrkC expressing cells that in turn could explain the absence or reduction of afferent projections. In the same way, we observed that, qualitatively, the innervation of the CGRP afferent fibers (TrkA-positive) in the hindpads of wild type animals is similar in Necdin adult mutant whereas the number of CGRP-positive nerves appears globally reduced. Again the loss of Trk A sensory neurons could explain this difference.

Previous *in vitro *experiments [11,22] suggest a function of Necdin in neurite outgrowth depending on NGF. In our mutant, we investigated whether, independently of the increase of apoptosis observed, the surviving sensory neurons could normally extend neurites in DRG explants cultures. We revealed a reduction in neurite outgrowth of E13.5 embryonic DRG mutant explants, dependant on NGF. This discrepancy between our *in vitro *results and our *in vivo *observations (no defects in axonal growth at E13.5) might be explained by a role of Necdin in late neuronal differentiation only. This Necdin function is separable from its anti-apoptotic role previously revealed. With this in mind, we report a loss of 27% of TrkA expressing cells at E13.5 and a 40% loss of TrkA expressing cells at P0, a substantial reduction between E13.5 and P0. Indeed, a role of Necdin in neurite outgrowth depending on NGF might explain this discrepancy: a deficiency in axonal outgrowth would then lead to an alteration of neuronal differentiation and an additional loss of TrkA cell death, in later embryogenesis stages. Furthermore, such a role would explain the observations reported by other groups [13,23].

### The Necdin anti-apoptotic function is not restricted to sensory neurons

In DRGs we observed an approximately 30% partial loss of all neurons. In previous work, such a loss has also been reported but not investigated in the hypothalamus of Necdin mutants (oxytocin- and LHRH-expressing neurons) [21]. More generally, we observed an increase of cell death in a whole Necdin embryo at an early stage of development (E10.5), as revealed by an anti-Caspase-3 labelling (Fig. 7B–C). This increase of apoptosis is not restricted to the nervous system but is extended to other tissues like somites or the extremities of members. An expression of Necdin has previously been reported in these structures [16].

Thus we propose that Necdin anti-apoptotic/survival function might be widespread in the nervous system and other tissues.

### Does the loss of sensory neurons explain the sensory defects observed in Necdin KO adult mice?

Sensory-motor studies on adult Necdin deficient mice showed sensory alterations (proprioceptive or nociceptive). These observations lead us to investigate the developing sensory neurons in DRGs. In accordance, we found cellular defects and revealed a loss of nociceptive (TrkA) and proprioceptive (TrkC) sensory neurons. However considering the cellular defects, we cannot immediately explain the sensory defects observed: from a loss of sensory TrkA cells, we would expect an increased pain threshold in Necdin deficient mice but we observe the opposite effect, (a higher sensitivity in the hot-plate test).

Furthermore from a loss of TrkC-cells, we would expect an alteration of the stretch reflex circuit with a diminution of the H-wave amplitude whereas, again, we observe the opposite effect with an increase of the H-wave amplitude only. A similar effect was observed in Amyotrophic Lateral Sclerosis disease in which a loss of motoneurons facilitates the stretch reflex circuit (increase of H-wave amplitude) [36]. In both cases, these data suggest either a hyper-excitability of sensory neurons or a lack of neuronal inhibition.

Thus to better understand the physiopathology of Necdin mutant mice, it will be necessary to take into account 1) all the cellular effect of Necdin in different neuronal populations and/or 2) the physiological compensation used by the organism to compensate for cellular impairment. Interestingly, recent data [37] suggest that Necdin promotes GABAergic differentiation; thus, a lack of GABAergic neurons in Ndn KO might lead a lack of neuronal inhibition.

## Conclusion

Our study reveals the sensory-motor behaviour of Necdin deficient mice. Overall, our data clarify the *in vivo *function of Necdin in growth arrest, survival and differentiation through neurogenesis. We show clearly that, in early development of the nervous system, Necdin is an anti-apoptotic or survival factor.

## Methods

### Breeding and genotyping of Necdin deficient Mice

Necdin deficient mice were generated as previously described [21]. We used a mouse colony generated on C57BL6 background (after10 backcross). Because *Necdin *is an imprinted gene, paternally expressed only, we crossed heterozygote males (+ m/- p) with wild type C57BL/6J females to produce experimental embryos; thus, in the generated litters, half the embryos were control and half were Necdin deficient. Age of embryos was determined by the presence of vaginal plug in the pregnant mothers and indicated as embryonic day 0.5. All embryos were genotyped by PCR with the primer set Nec4 (5'-TGC TAA GTG CCT ACA CTG AG-3') and Nec8 (5'-GCA TCT TAT TCA TGA GAG AC-3'); generating a 2.6 kb for the wild type allele and 1,2 kb fragment corresponding to the mutant allele.

### Immunohistochemistry

Embryos were collected, fixed and sectioned (cryostat, 10 or 12 μm). Antibodies used were: NC243 anti-mouse Necdin (rabbit polyclonal, 1/500, [38]), anti-Neurofilament (mouse monoclonal, 1:200, Chemicon); anti-TuJ1 (mouse monoclonal to neuron specific class III β-tubulin, 1:200, Berkeley Antibody); anti-Caspase-3-activated (rabbit polyclonal, 1/500, Upstate Biotechnology), anti-Runx3 (rabbit polyclonal, 1:200, a generous gift of Dr Yoran Gruner) anti-Parvalbumin (rabbit polyclonal antibody, 1/200, Swant Swisszerland) and anti-CGRP (Chemicon). Primary antibodies were used in a blocking solution containing 5% Goat serum, 1% BSA, 0.3% Triton-X-100. Biotinylated secondary antibodies and the ABC complex from the Vectastain kit (Vector Laboratories, Burlingame, CA) were used for detection. Alternatively, fluorophore-conjugated secondary antibodies were used (1/200; Jackson). Sections were examined on a Zeiss Axioplan 2 microscope with a CARV module. For whole-mount immunohistochemistry, individual embryos were processed in a whole-mount staining procedure using the monoclonal antibody TuJ1 or anti-Caspase-3-activated, as described [39].

### In situ hybridization

In situ hybridization (ISH) was carried out on 10 or 12-μ m cryosections and was performed as described previously [16]. The following in situ probes were used: *Trka, Trkb and Trkc *(a gift from Patapoutian)*, Islet *[40], *Ndn *[16], *p75NTR *(a gift from Dechant). Double in situ hybridization/immunohistochemistry was carried out as previously described [41]. Whole-mount ISH was carried out as previously described.

### BrdU labelling

In summary, pregnant mice were administered an intraperitoneal injection of 5-bromo-2'-deoxyuridine (BrdU, 50 mg/kg, Sigma) and sacrificed two hours later to allow BrdU incorporation into proliferating cells. Cryostat sections were post-fixed for 10 min in PFA 4%, washed in PBS, and denatured in HCL (2N) and triton-X-100 for 1 h at 37°C. The section was then neutralized for 10 min in sodium borate buffer (0,1 M, PH8.5) at room temperature. Immunohistochemistry was performed using anti BrdU monoclonal antibody diluted 1/2000 in PBS containing BSA 1%, NGS 5% and triton-X-100. Slides were incubated in primary antibody overnight at 4°C and detection was performed using anti-mouse FITC-conjugated secondary antibody (Jackson, 1:100).

### TUNEL assay

To identify cells undergoing apoptosis, terminal deoxynucleotidyl transferase-mediated biotinylated dUTP nick end labelling (TUNEL) technique was used. We performed TUNEL using the ApopTag Direct Kit (Oncor) and followed recommendations. To identify the proportion of neurons undergoing cell death in the early dorsal root ganglia of wild type and Necdin embryos, 10 or 12 μm frozen sections were doubly labeled for Neurofilament (NF), a neuron-specific marker, and TUNEL. Sections were first immunolabeled with mouse anti-NF monoclonal antibody (see above). This was followed by the TUNEL technique. The sections were examined and photographed using an Axioskop microscope. Cells undergoing apoptosis were recognized by an intensely fluorescent nucleus.

### Fluorojade staining

To detect any type of neuronal cell death, Fluorojade (FJ) labeling was performed on 10 μm thick frozen section as described previously [42].

### Measure of the length of nerves

Whole mount embryos were labeled with Tuj1 antibody as described above. Axonal outgrowth was measured at E12.5 (WT, n = 4; Ndn KO, n = 5) and E13.5 (WT, n = 7; Ndn KO, n = 9). Six peripheral axon bundles, corresponding to the lateral cutaneous branch were analyzed for each embryo and noted in micrometer.

### Explants culture

DRG explants were removed from E13.5 embryos and grown on coverslips coated with polylysin/laminin in Neurobasal medium containing 20% FBS and NGF (100 ng/ml). Ganglia were cultured for 20–24 hr then immunostained for Neurofilament prior to axon measurement. For each ganglion, the axonal surface was measured by subtracting nucleus area from the whole DRG area (nucleus + axon). For each animal, 10–12 ganglia were analyzed. A ratio was then constructed between the wild type and the mutant DRG.

### DRGs volume and cell count

Area of each DRG slide was calculated by using 12 μm serial section of L1 DRG at P0 from each genotype using the free UTHSCSA ImageTool program (developed at the University of Texas Health Science Center at San Antonio, Texas and available from the Internet by anonymous FTP from ). The DRG volume was then extrapolated and the values were noted in micrometer^3^.

Quantitative analysis to count the number of dying cells (TUNEL) or Trks and p75NTR positive cells was performed in at least 10 lumbar DRG sections per animal, and a mean number of positive cells per square millimeter was determined. All observations reported are based on analysis of multiple tissue sections from three to five Necdin mutant (paternal allele KO) and wild type mice from the same littermates.

### Statistical analysis

Statistical analyses were performed using appropriate nonparametric statistical tools (unpaired Mann-Whitney test). In the results, values are indicated as following: (Q2 (Q1, Q3), n). Q2 is the median and, between brackets, Q1 is first quartile and Q3 the second quartile. The level of significance was set at p < 0.05.

### Testing procedures

#### Gross neurological examination

The general health and basic sensory motor functions were evaluated using a modified SHIRPA protocol (EMPRESS, ). This analysis is adapted from that developed by Irwin [43] and from the SHIRPA protocol [44]. It provides an overview of physical appearance, body weight, body temperature, neurological reflexes and sensory abilities. Sensory functions were evaluated by measuring or scoring visual ability, audition, olfaction, tactile perception and vestibular function. Visual ability was assessed as orientation responses to an object (a white cotton swab) being moved in each peripheral visual field at a distance of 5 cm. Auditory function was evaluated by scoring Preyer and startle reflexes (pinna flicking backwards, startle) to 90 dB click noise of 20 kHz frequency. Olfaction was evaluated by scoring olfactory exploration of an object (a cotton swab) presented in front of the animal's muzzle. Tactile perception was evaluated by scoring the mouse reaction to pinna and corneal touch using a cotton wire. Vestibular function was evaluated by measuring righting and contact righting reflexes.

#### Rotarod test

This test measures the ability of an animal to maintain balance on a rotating rod (Bioseb, Chaville, France). This task requires a variety of proprioceptive, vestibular and fine-tuned motor abilities. Mice were first given three habituation trials for 60 sec each (one trial at 0 rpm and 2 trials at 4 rpm). Animals were then submitted to 4 testing trials separated by 5–10 min interval and during which the rotation speed accelerated from 4 to 40 rpm in 5 min.

#### Beam walking

The beam walking test is used to evaluate fine motor coordination and proprioceptive function, requiring accurate paw placement [45]. The apparatus used is a 2 cm diameter and 110 cm long wooden beam, elevated 50 cm above the ground. The beam is subdivided into 11 segments allowing rapid estimation of the distance crossed by the mouse. The first segment at one extremity of the beam is used as a starting point during testing trials. A goal box (12 × 12 × 14 cm) is fixed at the other extremity of the beam.

Animals are first habituated to the goal box for 1 min. They are then submitted to 3 training trials during which they are placed at different points of the beam the head directed to the goal box and allowed to walk the corresponding distance to enter the goal box. After training, animals are submitted to 3 testing trials during which they are placed at the extremity of the beam opposite to the goal box and allowed to walk the beam distance and enter the goal box. The latency to enter the goal box and the number of slips (when one or both hindpaws slips laterally from the beam) are measured.

#### String test (traction reflex test)

The apparatus is a wire stretched horizontally 40 cm above a table. Testing consists of 3 consecutive trials separated by 5–10 min interval. On each trial the forepaws of the animal are placed on the thread. The latency the animal took to catch the wire with its hindpaws was recorded [46].

#### Grip test

This test measures the maximal muscle strength using an isometric dynamometer connected to a grid (Bioseb). Once the animal is holding the grid with its all paws it is slowly moved backwards until it releases it. The dynamometer records the maximal strength developed.

#### Tail flick test

This apparatus consists of a shutter-controlled lamp as a heat source (Bioseb). Three consecutive trials with an interval of about 1–2 min are performed at different sites of the tail. For each trial, the tail of the animal is placed under the heat source. The time taken by the animal to flick its tail is recorded (cut off 20 s).

#### Hotplate test

The mice are placed into a glass cylinder on a hot plate adjusted to 52°C (Bioseb) and the latency of the first reaction (licking, moving the paws,) or jump is recorded. Two consecutive trials with an interval of about 15 min are performed. The first trial ends either immediately after the mouse displays the first reaction or after 30 s if it does not show any sign of pain. The second trial ends either immediately after the mouse displays a jump or after 3 min if it does not show any jump.

#### Electromyography

EMG recordings were performed under Ketamine-xylazine anaesthesia using a Key Point electromyograph apparatus (Medtronic, France). The body temperature is maintained at 37°C with a homeothermic blanket (Harvard, Paris, France).

- For measuring SNCV, recording electrodes are inserted at the base of the tail and stimulating electrodes placed 20 mm from the recording needles towards the extremity of the tail. A ground needle electrode is inserted between the stimulating and recording needles. Caudal nerve is stimulated with a series of 20 pulses during 0.2 ms each at a supra-maximal intensity. The mean response of these 20 stimulations is included for statistical analysis.

- CMAP was measured in gastrocnemius or plantar muscle after stimulation of the sciatic nerve. For this purpose, stimulating electrodes are placed at the level of the sciatic nerve and recording electrodes are placed in the gastrocnemius or plantar muscle. A ground needle is inserted in either the contralateral paw. Sciatic nerve is stimulated with a single 0.2 ms pulse at a supra-maximal intensity. The amplitude (mV) and the distal latency (ms) of the responses are measured.

#### The open field test

*T*he openfield test allows evaluation of anxiety and exploratory drive. Mice are tested in automated open fields (Panlab, Barcelona, Spain). The open field arena is divided into central and peripheral regions. The open fields are placed in a room homogeneously illuminated at 150 Lux. Each mouse is placed in the periphery of the open field and allowed to explore freely the apparatus for 30 min, with the experimenter out of the animal's sight. The distance travelled, the number of rearing, and time spent in the central and peripheral regions are recorded over the test session. The latency and number of crosses into as well as the percent time spent in center area are used as indices of emotionality/anxiety

#### Statistical analysis

statistical analyses were performed using appropriate nonparametric statistical tools: mainly unpaired Mann-Whitney test. Qualitative parameters (e.g. clinical observations) were analyzed using χ^2 ^test. The level of significance was set at p < 0.05.

## Authors' contributions

DA carried out all the *in vivo *cellular studies, made the figures and participated in the draft of the manuscript. HM carried out the behaviour analysis and participated in the draft of the manuscript, concerning the behavioural data. F. Marly carried out the CGRP study. CA carried out the DRG explants cultures. PAF carried out the first in vivo cellular studies with DA. FM conceived the study, participated in its design, coordination and drafted out the manuscript. All authors read and approved the final manuscript.

## Supplementary Material

Additional File 1 Comparative quantification of TrkA, TrkB and TrkC expressing cells in the lumbar and thoracic DRGs between wild type and mutant E13.5 embryos. The data provided represent the comparative quantification of TrkA, TrkB or TrkC expressing cells in the lumbar (A) and thoracic (B) DRGs between wild-type and mutant E13.5 embryos. Shown are the mean numbers of neurons ± SEM per square millimeter. Statistical comparisons were made using the Mann-Whitney test; asterisks show differences that are statistically significant (*, p < 0.05) Click here for file

Additional File 2 Reduction of the number of TrkA- and TrkC-expressing cell in Necdin mutant trigeminal ganglia. The data provided represent the density of *Trk*-expressing neuron in trigeminal ganglia at E13.5. Box-plots are representative of neuronal counts from five or more embryos. Significant loss of TrkA- and TrkC- and no lack of TrkB-cells number were observed. Statistical comparisons were made using the Mann-Whitney test; asterisks show differences that are statistically significant (*, p < 0.05). Click here for file
