# Supplementary figures and images for: Sensory defects in Necdin deficient mice result from a loss of sensory neurons correlated within an increase of developmental programmed cell death
Source: BMC Dev Biol. 2006 Nov 20;6:56. doi: 10.1186/1471-213X-6-56 (PMC1687209; doi:10.1186/1471-213X-6-56)

## Additional file 1

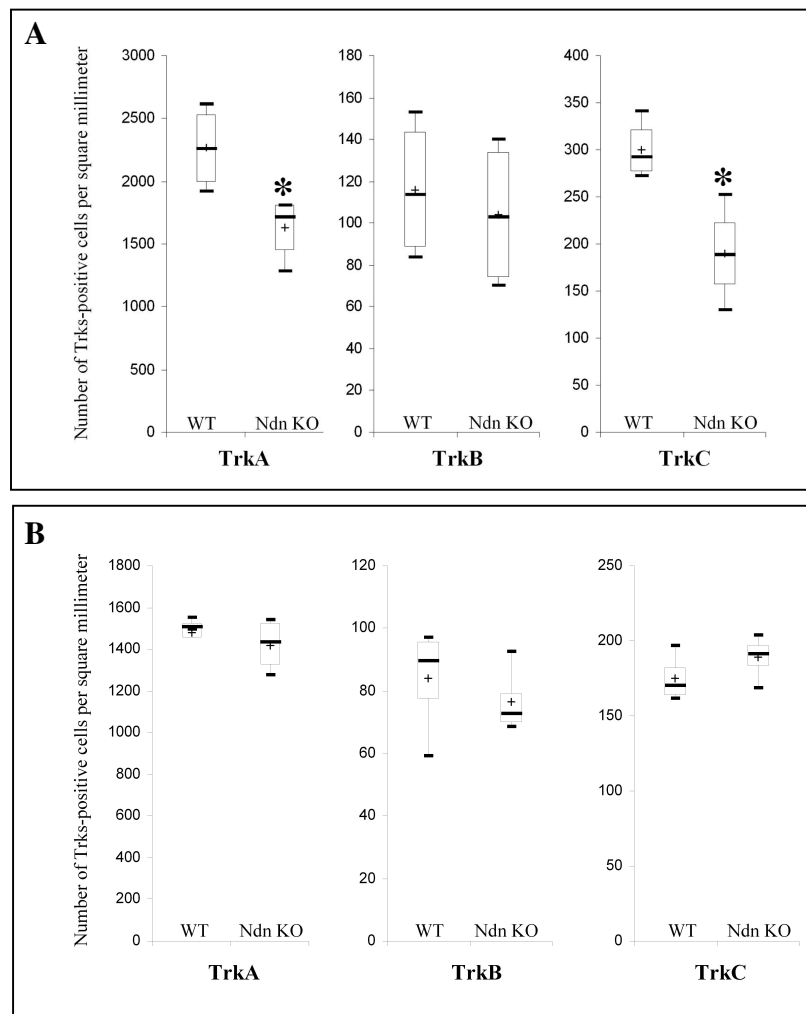

## Additional file 2

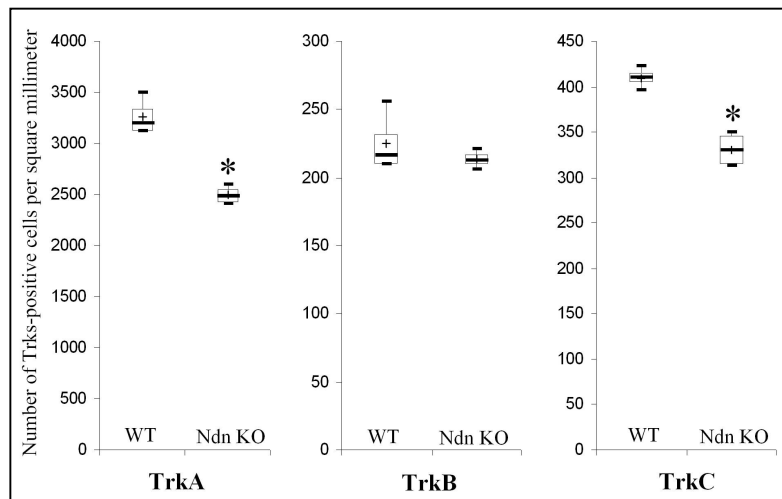

Supplement: Additional File 1 — Comparative quantification of TrkA, TrkB and TrkC expressing cells in the lumbar and thoracic DRGs between wild type and mutant E13.5 embryos. The data provided represent the comparative quantification of TrkA, TrkB or TrkC expressing cells in the lumbar (A) and thoracic (B) DRGs between wild-type and mutant E13.5 embryos. Shown are the mean numbers of neurons ± SEM per square millimeter. Statistical comparisons were made using the Mann-Whitney test; asterisks show differences that are statistically significant (*, p < 0.05) [file 1471-213X-6-56-S1.pdf]
